# Percutaneous electric nerve field stimulation alters cortical thickness in a pilot study of veterans with fibromyalgia

**DOI:** 10.1016/j.ynpai.2022.100093

**Published:** 2022-05-17

**Authors:** Anna Woodbury, Lisa C. Krishnamurthy, Anastasia Bohsali, Venkatagiri Krishnamurthy, Jeremy L. Smith, Melat Gebre, Kari Tyler, Mark Vernon, Bruce Crosson, Jerry P. Kalangara, Vitaly Napadow, Jason W. Allen, Daniel Harper

**Affiliations:** a Emory University School of Medicine, Atlanta, GA, USA; b Atlanta Veterans Affairs Healthcare System, Atlanta, GA, USA; c Georgia State University, Atlanta, GA, USA; d Spaulding Rehabilitation Network, Harvard Medical School, Charlestown, MA, USA

**Keywords:** Fibromyalgia, Pain, Percutaneous Electric, Nerve Stimulation, Neuroimaging, Veterans

## Abstract

**Objective:**

To evaluate changes in cortical thickness and right posterior insula (r-pIns) gamma-aminobutyric acid (GABA) concentrations in veterans with fibromyalgia treated with auricular percutaneous electric nerve field stimulation (PENFS).

**Materials & methods:**

This was a randomized, controlled, open label investigation conducted in a government hospital. Twenty-one veterans with fibromyalgia were randomized to receive either standard therapy (ST; i.e., 4 weekly visits with a pain practitioner) or ST with auricular PENFS (ST + PENFS). Neuroimaging data was collected at baseline (i.e. before the first treatment session) and again within 2 weeks post-treatment.​ Clinical pain and physical function were also assessed at these timepoints. Single-voxel magnetic resonance spectroscopy was carried out in r-pIns to assess changes in r-pIns GABA concentrations and high-resolution T1-weighted images were collected to assess changes in regional gray matter volume using cortical thickness.

**Results:**

Both the ST + PENFS and ST groups reported a decrease in pain with treatment. **Volumetric:** Cortical thickness significantly decreased in the left middle posterior cingulate (p = 0.018) and increased in the left cuneus (p = 0.014) following ST + PENFS treatment. These findings were significant following FDR correction for multiple comparisons. ST group right hemisphere insula cortical thickness increased post-treatment and was significantly (p = 0.02) inversely correlated with pain scores. ST + PENFS group right hemisphere posterior dorsal cingulate size significantly (p = 0.044) positively correlated with pain scores. **GABA**: There were no significant correlations with GABA, though a trend was noted towards increased GABA following treatment in both groups (p = 0.083) using a linear mixed effects model.

**Conclusions:**

Results suggest a novel effect of PENFS reflected by differential volumetric changes compared to ST. The changes in GABA that occur in both groups are more likely related to ST. Insular GABA and cortical thickness in key regions of interest may be developed as potential biomarkers for evaluating chronic pain pathology and treatment outcomes.

## Highlights

• Fibromyalgia pain improved following treatment with standard therapy or PENFS.
• Right insular increases in cortical thickness correlated with improved pain scores.
• Posterior dorsal cingulate cortical thickness decreased with improved pain scores.
• Cortical thickness decreased in the left middle posterior cingulate following PENFS.
• Cortical thickness decreased in the left cuneus following PENFS.

## Graphical abstract

**Figure:**
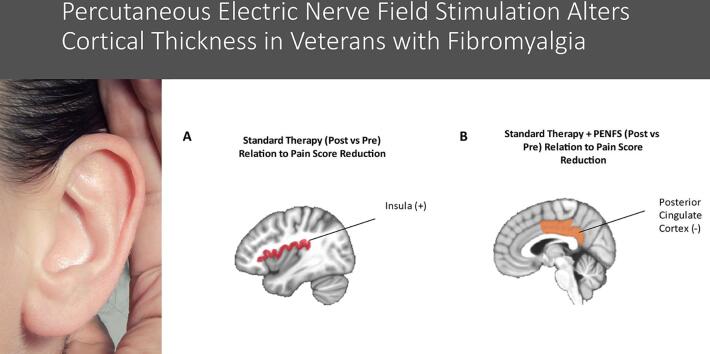

## Introduction

Our group is interested in the neurological underpinnings of pain perception and investigating improved treatments for chronic pain management. Fibromyalgia is a chronic pain condition involving spontaneous widespread pain and fatigue (Clauw, 2014)and affects millions of people worldwide. It is often accompanied by significant decreases in quality of life for the affected individuals, and it presents an economic burden to the healthcare system. (Heidari et al., 2017, Spaeth, 2009) Still, there is no scientific consensus on the etiological underpinnings of the condition, although decades of research have implicated central nervous system abnormalities in patients with fibromyalgia.

Previous studies have demonstrated that fibromyalgia is associated with volumetric changes in regional gray matter (consistent decreases in bilateral anterior cingulate cortex, paracingulate cortex, medial prefrontal cortex, and posterior cingulate/paracingulate cortex; left parahippocampal gyrus and fusiform cortex; and right parahippocampal gyrus and hippocampus; and consistent increases in the left cerebellum). (Pomares et al., 2017, Puiu et al., 2016, Shi et al., 2016) Differences in gamma-aminobutyric acid (GABA), the brain’s main inhibitory transmitter system, when compared to individuals without chronic pain also have been found. (Foerster et al., 2012, Harris et al., 2013, Pomares et al., 2020, Legarreta et al., 2021) In combat veterans, gray matter changes have been noted in association with chronic pain; changes in cortical thickness in the left inferior frontal gyrus, superior parietal cortex, right rostral middle frontal gyrus, precentral and postcentral gyri, and superior temporal cortex have been negatively associated with combat exposure in veterans with chronic pain, suggesting that chronic pain may modulate the relationship between combat stress and cortical thickness. (Corbo et al., 2016) We therefore evaluated cortical thickness in these brain regions that have been previously implicated in fibromyalgia and chronic pain to evaluate whether pain treatment could acutely alter gray matter. We targeted the right posterior insula (r-pIns) for evaluation of GABA changes based on prior literature indicating changes in GABA were related to changes in pain for fibromyalgia. (Foerster et al., 2012, Harris et al., 2013).

In the present study, we investigated the effect of auricular percutaneous electrical nerve field stimulation (PENFS) using a FDA-approved device for pain that delivers stimulation to the auricular branches of the cranial nerves, including the vagus nerve, in veterans with fibromyalgia. More specifically, we examined the impact of PENFS and standard therapy (ST) on 1) cortical gray matter thickness in pre-determined regions of interest (ROIs) as stated above and 2) GABA concentrations in the right posterior insula (r-pIns) and correlated these neuroimaging findings with behavioral outcomes and pain scores as rated by the Defense and Veterans Pain Rating Scale (DVPRS). The overarching goals of this pilot study were 1) to evaluate PENFS as an adjunct treatment for fibromyalgia compared to standard therapy alone and 2) to further develop potential structural and chemical neuroimaging biomarkers for fibromyalgia and its management.

## Materials & methods

### General procedures

This was a feasibility study in which only the outcomes assessor was blinded. Methods pertaining to participant screening and recruitment, magnetic resonance imaging (MRI) acquisition, randomization, intervention, behavioral outcome measures and follow-up are further detailed in our prior publications related to evaluation of resting state functional connectivity MRI (rs-fcMRI) in treatment of fibromyalgia with PENFS (Military Field Stimulator©, Innovative Health Solutions, Versailles, IN, USA). (Woodbury et al., 2020, Gebre et al., 2018) Participants were allowed to use standard therapy based on individual preferences, comorbidities, and recommendations from their clinical care provider. To control for the influence of weekly visits in the PENFS group, participants in the ST only group were also provided weekly visits with medication increases as needed for pain during their 4-week study treatment period. The study was conducted in accordance with ethical principles from the Declaration of Helsinki and the Ethical Committee at Karolinska Institute and approved by the Institutional Review Board of Emory University and the Veterans Affairs Research & Development committee.

### Study participants

**Fig. 1:**
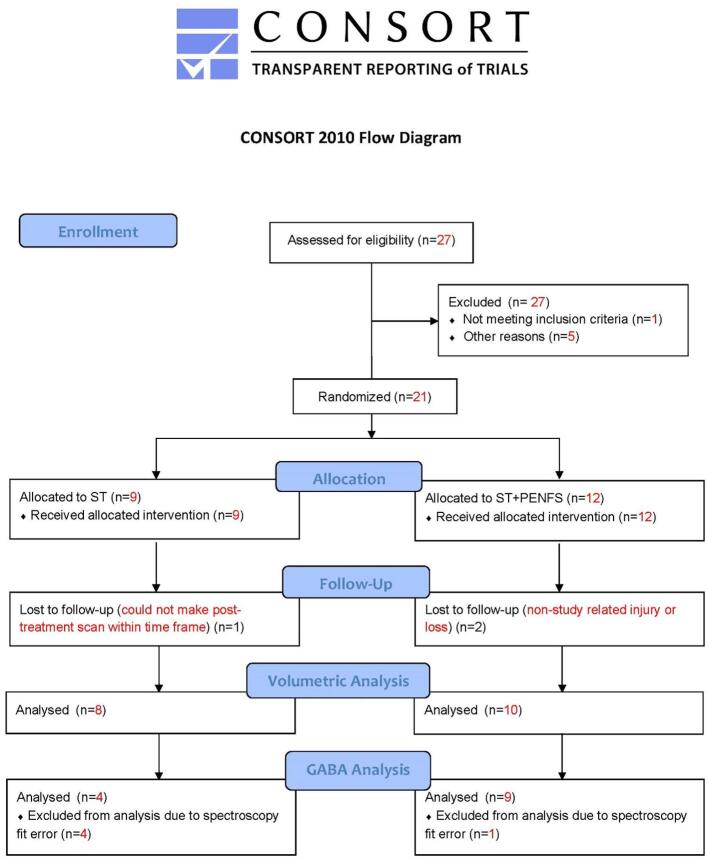
Consort Diagram for Cortical Thickness & GABA Analysis.

In brief, 21 veterans aged 20–60 yrs who met American College of Rheumatology 2010 (Sarzi-Puttini et al., 2018) criteria for fibromyalgia and were able to safely tolerate MRI were block-randomized, stratified for age and sex, to receive either ST (n = 9) or PENFS treatment in addition to ST (n = 12).Veterans were recruited from the pain clinic and referred from their clinical providers. Veterans who qualified were invited via phone calls to participate in the study. Twenty-seven participants were screened, 6 excluded, 21 randomized (9 to ST and 12 to ST with PENFS), and3 lost to follow-up (Fig. 1). All subjects provided written informed consent.

### Application of PENFS

PENFS was applied to the external ear by a board-certified pain physician using an FDA-cleared neuromodulating generator with the following settings: frequency 1–10 Hz, pulse width 1 ms, amplitude 3.2 v, impulse 100 mw, length of stimulation 120 hrs, duty cycle of 2 hrs on / 2 hrs off. Neither the provider nor the participant was blinded to the intervention. Participants went home with the device, which provides continuous stimulation for 5 days, after which the device was exchanged. This procedure was completed weekly over 4 weeks and is further described in prior publications. (Woodbury et al., 2020, Gebre et al., 2018).

### Assessment of pain rating

DVPRS (Nassif et al., 2015) was administered at each of 3 visits (baseline, 6 weeks and 12 weeks after initiation of therapy) to monitor changes in pain during participant enrollment. The DVPRS includes a validated pain assessment that combines the visual analogue scale (VAS), numerical pain rating scale (NPRS), verbal rating scale (VRS), and the FACES scale, to apply to participants of various cognitive abilities. The self-reported pain level circled by the participant was scaled on the NPRS portion. Additionally, participants were asked to answer 4 pain supplemental questions on the DVPRS related to pain interference with activity, sleep, mood, and stress, which are further detailed in our prior publication. (Woodbury et al., 2020).

### MRI data acquisition

MRI acquisition was performed at baseline, 2 weeks prior to initial therapy (‘pre’) and 2 weeks following the completion of a 4-week treatment period (‘post’). To quantify cortical thickness for the whole brain, a high resolution T1w anatomical scan was collected: sagittal 3D MPRAGE acquisition, TR = 2300 ms, TE = 2.89 ms, TI = 800 ms, FA = 8 deg, FOV = 256 × 256 mm^2^, 176 slices, voxel size = 1x1x1 mm^3^, acquisition bandwidth = 140 Hz/px, total scan duration = 9:50 min. Acceleration techniques such as phase or slice partial Fourier or GRAPPA were not utilized to ensure high signal to noise ratio (SNR), as interpolation of k-space can blur the image and increase uncertainty in the cortical thickness quantification. The images were spot-checked immediately after the acquisition for motion artifacts and recollected if present.

### GABA MRS data acquisition

During the MRI scan, immediately after the acquisition of the T1w anatomical scan, Magnetic Resonance Spectroscopy (MRS) was performed to evaluate GABA concentrations in the right posterior insula (r-pIns). The J-edited (Frangos et al., 2015) MRS acquisition utilized the Center for Magnetic Resonance Research (CMRR) Spectroscopy Tools Mescher-Garwood Point Resolved Spectroscopy (MEGA-PRESS) (Zhu et al., 2021) sequence to separate the small GABA + signals from the rest of the MR spectrum (TR = 2000 ms, TE = 68 ms, voxel size = 3x3x3 cm^3^, acquisition bandwidth = 2000 Hz, acquisition duration = 1024 ms, vector size = 2048, VAPOR water suppression bandwidth = 135 Hz, editing pulse bandwidth = 53 Hz, ON editing pulse = 1.9 ppm, OFF editing pulse = 7.5 ppm, total scan duration = 10 min,). Each free induction decay (FID) was collected and stored separately for use in preprocessing. The CMRR Spectroscopy Tools FAST(EST)MAP (Grayston et al., 2019, Perini et al., 2017) was used to achieve a high-quality shim in the r-pIns. The voxel was placed in r-pIns on the high resolution T1w MPRAGE described above by trained study personnel. The r-pIns was chosen for this experimental paradigm because of prior literature indicating changes in GABA in the r-pIns related to pain therapy in fibromyalgia. (Foerster et al., 2012, Harris et al., 2013) An unsuppressed water (H_2_O) spectrum with matching acquisition parameters was also collected from the same region.

A 3x3x3 cm GABA voxel was placed in the r-pIns in each subject for MRS acquisition with concurrent structural and rs-fcMRI acquisition. (Gebre et al., 2018).

### Cortical thickness analysis

Volumetric assessments for gray matter cortical thickness were performed using Freesurfer on high-resolution T1-weighted images. Twenty-one pre-processed (Woodbury et al., 2020) Freesurfer (Cardinale et al., 2014, Magon et al., 2018) datasets were quality-controlled; three datasets from the PENFS group were excluded from analysis due to missing datapoints or circumstances that affected participants’ behavioral measures; two datasets from the ST group were excluded due to missing datapoints. PENFS and standard therapy participants’ data along with demographics and pain measures were compiled into a general linear model following the paired group analysis in Freesurfer. The following group comparisons were performed: (1) voxel-wise whole brain comparison of cortical thickness for ‘post’ vs ‘pre’ scanning sessions; (2) voxel-wise whole brain correlations of cortical thickness changes and changes in pain scores for ‘post’ vs ‘pre’ scanning sessions. Whole brain cortical thickness data was smoothed with a 5 mm Gaussian smoothing kernel to account for individual differences in gyral and sulcal anatomy. Comparisons were made for twelve ROIs and whole brain vertex analysis. ROI-based cortical parcellations were not smoothed. To correct the ROI-based volume/cortical thickness analyses for multiple comparisons we used Matlab’s estimate of positive false discovery rate (FDR) for multiple hypothesis testing for ‘post’ vs ‘pre’ comparisons and p < 0.05/2 = 0.025 for regression analyses.

Regions of interest included cortices of left and right hemisphere anterior cingulate, insula, fusiform gyrus, parahippocampal gyrus, cuneus, middle frontal gyrus, angular gyrus, precentral gyrus, postcentral gyrus, precuneus, middle temporal gyrus, & volumetric cerebellum and hippocampus identified based on evidence from existing literature related to fibromyalgia and gray matter alterations. (Pomares et al., 2017, Puiu et al., 2016, Shi et al., 2016).

### GABA magnetic resonance Spectroscopy (MRS) analysis

MRS data was pre-processed in the GABA Analysis Toolkit (Gannet, version 3.1.4: gabamrs.com), a MATLAB-based toolbox designed for analysis of difference-edited spectra and Mescher-Garwood (MEGA)-PRESS acquisitions in particular. (Edden et al., 2014, Harris et al., 2015, Near et al., 2013) For Siemens data, Gannet’s processing pipeline consists of frequency and phase correction in the time domain, line broadening using an exponential apodization function, and secondary frequency and phase correction using choline and creatine signal fits. Following these corrections, the program outputs the edited difference spectrum. The MRS voxel is then co-registered to the subject’s T1-weighted anatomical image, which is used to derive gray matter, white matter, and CSF voxel fractions to output tissue-corrected GABA concentrations.

Following Gannet processing, subjects with creatine FWHM ≤ 14 Hz and GABA fit error ≤ 9% were retained. Upon visual inspection, spectroscopic voxel placement (MNI coordinates: 38, −14, 7) in the r-pIns was confirmed to be consistent across subjects, although Gannet estimates of voxel tissue fractions indicated that one acquisition had included a larger ratio of white to grey matter than others, and this subject was excluded from further analysis. An additional subject was excluded as an outlier based on creatine AUC. Including these two subjects, a total of 5 subjects were excluded due to spectroscopy fit error (Fig. 1). With these exclusions, the final cohort included 4 ST control (8 acquisitions, mean age 49.3 ± 12.4 years) and 9 ST + PENFS (8 acquisitions, mean age 51.8 ± 8.9 years) individuals. The final cohort did not differ with respect to metabolite SNR or fit error, creatine or NAA FWHM, or tissue fraction as a function of *treatment* (ST vs. ST + PENFS), *scan* (baseline vs. week 4), or *treatment* × *scan* interaction at p > 0.05. Due to the small sample size, however, subjects randomized to ST + PENFS treatment did exhibit lower DVPRS scores at baseline than the control group [F(1, 13) = 7.419, p = 0.017, η2partial = 0.363].

Cortical thickness measurements were analyzed in ROIs related to pain in ST and ST + PENFS groups, revealing significant differences following treatment, though pain scores did not significantly differ. Results of the analyses are described with and without FDR correction for multiple comparisons, as this is a pilot study intended to generate hypotheses for future investigations. Laterality is reported based on theoretical differences in emotion and pain processing between the two hemispheres. (Pauli et al., 1999).

### ST (Control) Cortical Thickness (Post-Pre)

After correcting for multiple comparisons, ROI-based analysis did not reveal any regions with significant ‘post’ vs ‘pre’ cortical thickness differences for the ST control group. However, prior to correcting for multiple comparisons, three regions, namely, left middle frontal gyrus (p = 0.041), left parahippocampal gyrus (p = 0.033), and left precuneus (p = 0.003), showed significant increases for the ‘post’ vs ‘pre’ cortical thickness measures prior to multiple comparison correction for the ST control group. Right hippocampal cortical thickness also significantly increased (p = 0.036) between the ‘post’ and ‘pre’ sessions for the ST control group prior to multiple comparison correction (Table 1).

**Table 1:** Changes in Cortical Thickness Following Treatment. Regions of interest (ROI) were analyzed for cortical thickness changes from pre- to post- treatment. Left- and right-hemisphere areas were both analyzed using paired *t*-test and FDR correction for multiple comparisons. Averaged data for left hemisphere cortical thickness significantly (p < 0.05) decreased in the middle posterior cingulate increased in the cuneus following ST + PENFS. These findings were significant following FDR correction for multiple comparisons. Significant findings in the control group did not survive multiple comparisons corrections. Uncorrected p-values are presented in the table; FDR corrected p-values are in parentheses.

Post vs. Pre Cortical Thickness Comparison				
Standard Therapy (Control)	Region of Interest (Hemisphere)		T-stat	p-value uncorrected (corrected)
	Middle frontal gyrus	Left:	2.086	0.041*
		Right:	1.835	0.058
	Parahippocampal gyrus	Left:	2.247	0.033*
		Right:	1.735	0.067
	Precuneus	Left:	4.014	0.003*
		Right:	−0.537	0.305
	Hippocampus	Left:	0.791	0.229
		Right:	2.19	0.036*
Standard Therapy + PENFS (Intervention)	Region of Interest (Hemisphere)		T-stat	p-value
	Posterior cingulate	Left:	−2.592	0.018*(0.022) ^¥^
		Right:	0.531	0.306
	Cuneus	Left:	2.786	0.014*(0.033) ^¥^
		Right:	−1.139	0.146
	Central insula	Left:	−2.134	0.035*
		Right:	−1.668	0.07
	Angular gyrus	Left:	−1.051	0.164
		Right:	−3.844	0.003*
	Precuneus	Left:	−0.577	0.291
		Right:	−1.881	0.051*

* uncorrected, significant; ^¥^significant after FDR correction for multiple comparisons.

### ST + PENFS Treatment Cortical Thickness (Post-Pre)

**Fig. 2:**
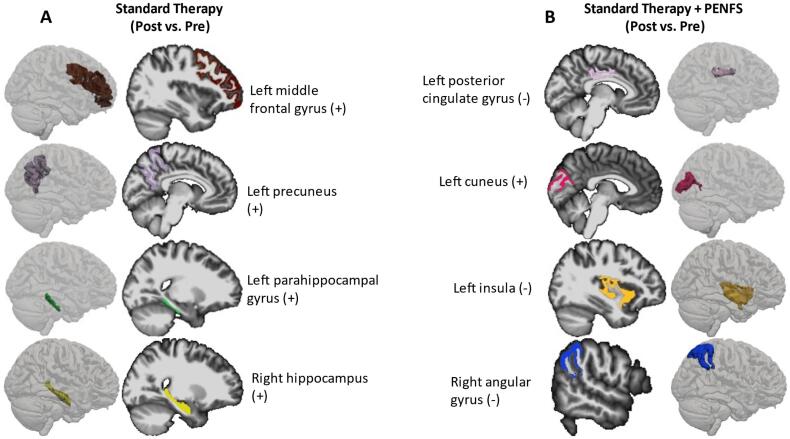
Changes in cortical thickness associated with standard therapy (ST) and ST + PENFS. The cortices for the regions of interest (ROI) with significant correlations are depicted both in 3 dimensions and with a 2-dimensional sagittal slice extracted from FreeSurfer. (+) denotes an increase in cortical thickness for the ROI following treatment. (-) denotes a decrease in cortical thickness following treatment. A. Cortical thickness for left middle frontal gyrus, precuneus and parahippocampal gyrus increased with standard therapy compared to baseline. These findings did not survive FDR correction for multiple comparisons. B. Cortical thickness in the left hemisphere significantly (p < 0.05) decreased in the middle posterior cingulate and increased in the cuneus following ST + PENFS compared to baseline. These findings were significant following FDR correction for multiple comparisons. Left central insula and right angular gyrus cortical thickness significantly (p < 0.05) decreased as well, though these findings were not significant following FDR correction for multiple comparisons.

ROI-based analysis revealed two regions in the left hemisphere, namely, middle posterior cingulate gyrus and sulcus (p = 0.018; decreased) and cuneus (p = 0.014; increased) with significant ‘post’ vs ‘pre’ cortical thickness difference for the ST + PENFS group after correcting for multiple comparisons. Additional areas trended towards significance but failed to remain significant after corrections for multiple comparisons. These regions with significant decreases in ‘post’ vs ‘pre’ cortical thickness prior to correction for multiple comparisons were: left insula (p = 0.035), right precuneus (p = 0.05), and right angular gyrus (p = 0.003) (Table 1**,** Fig. 2).

### Cortical thickness and pain score correlations

After correcting for multiple comparisons, ROI-based regression analysis did not reveal any regions with significant (p < 0.003) correlations between the ‘post’ vs ‘pre’ cortical thickness differences and ‘post’ vs ‘pre’ pain score differences for the ST control group. However, prior to correction for multiple comparisons, the right insula cortical thickness significantly correlated with pain scores (p = 0.02, adj. R2 = 0.556), with thickness increasing as pain scores decreased for ‘post’ vs ‘pre’ scanning sessions for the ST control group.

**Fig. 3:**
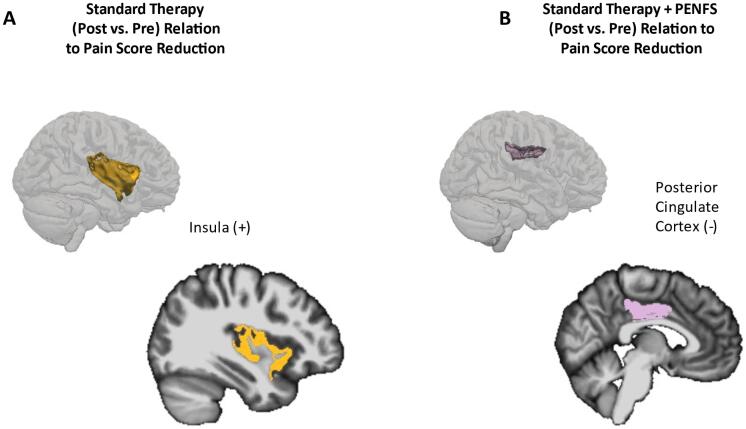
Changes in cortical thickness of right insula and posterior dorsal cingulate cortex associated with improved pain score. The cortices for the regions of interest (ROI) with significant correlations to pain scores are depicted both in 3 dimensions and with a 2-dimensional sagittal slice extracted from FreeSurfer. (+) denotes an increase in cortical thickness for the ROI following treatment. (-) denotes a decrease in cortical thickness following treatment. A. Standard therapy group right hemisphere insula size significantly (p < 0.05) inversely correlated with pain scores; as insular size increased, pain scores decreased. These findings did not survive FDR correction for multiple comparisons. B. ST + PENFS group right hemisphere posterior dorsal cingulate size significantly (p < 0.05) positively correlated with pain scores; as posterior dorsal cingulate size decreased, pain scores decreased. These findings also did not survive FDR correction for multiple comparisons.

ROI-based regression analysis did not reveal any regions with significant (p < 0.003) ‘post’ vs ‘pre’ cortical thickness differences and ‘post’ vs ‘pre’ pain score differences for the ST + PENFS group (this includes before and after correction for multiple comparisons) for the left hemisphere. ROI-based regression analysis did not reveal any regions with significant (p < 0.003) ‘post’ vs ‘pre’ cortical thickness differences and ‘post’ vs ‘pre’ pain score differences for the ST + PENFS group after correcting for multiple comparisons for the right hemisphere. There was one region, namely, the right posterior dorsal cingulate, that showed significant correlation (adj. R2 = 0.385, p = 0.044) between cortical thickness and pain scores for ‘post’ vs ‘pre’ scanning sessions for the ST + PENFS group prior to correction for multiple comparisons. Thus, as pain scores decreased, so did cortical thickness. Left and right cerebellar volumes as well as left and right hippocampal volumes did not show significant correlations with pain scores (Table 2**,** Fig. 3).

**Table 2:** Correlations Between Changes in Cortical Thickness and Pain Scores. Standard therapy group right hemisphere insula size significantly (p < 0.05) inversely correlated with pain scores; as insular size increased, pain scores decreased. ST + PENFS group right hemisphere posterior dorsal cingulate size significantly (p < 0.05) positively correlated with pain scores; as posterior dorsal cingulate size decreased, pain scores decreased.

Post vs Pre Cortical Thickness and Pain Scores Regression			
Standard Therapy (Control)	Region of Interest	T-stat	p-value
	Right Insula	−3.127	0.02
Standard Therapy + PENFS (Intervention)	Posterior dorsal cingulate	2.45	0.044

### GABA changes with treatment over time

**Fig. 4:**
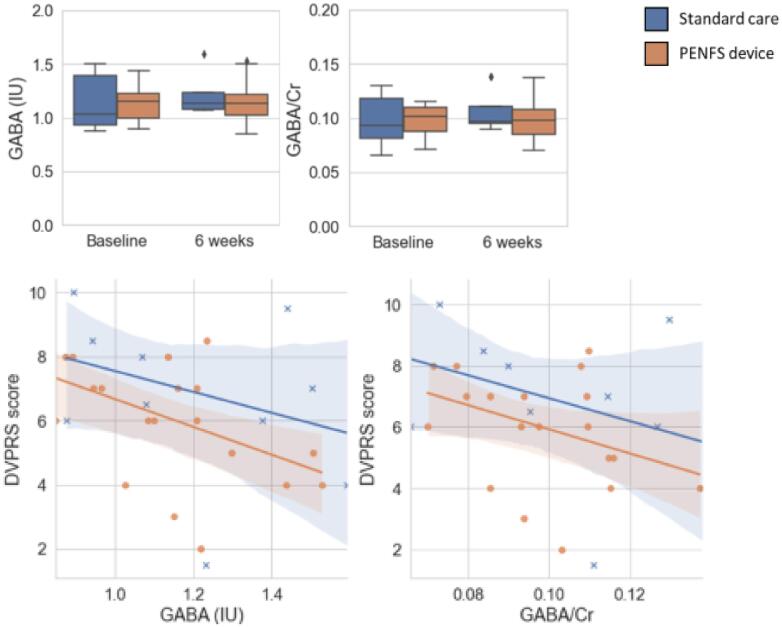
Changes in GABA Related to Changes in Pain on DVPRS. Changes in GABA (IU, CSF corrected) and GABA/Cr ratios are denoted at baseline and 6 weeks. GABA values from all scans are plotted on the scatterplots against pain scores, as measured by the DVPRS.

When GABA values (IU, CSF corrected) and GABA/Cr ratios were correlated with pain scores, no significant changes were noted (Fig. 4). There was generally an inverse correlation between GABA in the r-pIns and DVPRS pain scores that occurred with both groups (p = 0.083). Across all scans at all timepoints, as GABA concentrations in the r-pIns increased, pain scores decreased.

## Conclusions

We have previously reported a positive effect of PENFS on pain when compared to standard therapy alone; (Woodbury et al., 2020) results of our current analysis suggest a neural correlate for these treatment effects in terms of changes in cortical thickness after 4 weeks of treatment. While initial results concerning this non-pharmacologic treatment for fibromyalgia are promising, the clinical efficacy of PENFS for fibromyalgia should be explored in larger, randomized, double-blind, placebo-controlled trials. Furthermore, these interesting albeit preliminary neuroimaging outcomes should be evaluated not only in larger samples for confirmatory evidence of these relationships, but also at later time-points to evaluate the long-term neuromodulatory effects of PENFS.

Given the established role of the r-pIns in fibromyalgia-related pain, we evaluated the insula both using GABA MRS as well as cortical thickness assessments. Our GABA analysis was limited by a small number of MRI acquisitions meeting criteria for GABA spectroscopy fit error (n = 9 for PENFS with ST, and n = 4 for ST alone; Fig. 1). Thus, we can make only very general statements regarding the overall findings and are unable to assess group differences. Although not significant, DVPRS measures were inversely correlated with GABA (IU) (Fig. 4). This is consistent with prior literature, in which insular GABA levels increase as pain scores decrease.^5,8^.

Pain scores and cortical thickness were correlated using regression analysis, resulting in significant findings. The averaged standard therapy group right hemisphere insula thickness significantly (p = 0.02) and inversely correlated with pain scores; as insular size increased, pain scores decreased, whereas in the PENFS group, right hemisphere posterior dorsal cingulate size significantly (p = 0.044) positively correlated with pain scores; as posterior dorsal cingulate size decreased, pain scores decreased. The dorsal posterior cingulate cortex is a central node of the default mode network and has also been implicated in pain (Magon et al., 2018). It is interesting that acute changes can be found immediately post-treatment in cortical thickness values, but it is even more noteworthy that these areas differ between groups treated with standard therapy vs. those treated with standard therapy and the addition of PENFS.

Several cortical areas that were previously implicated in connectivity (Woodbury et al., 2020) to the r-pIns were found in the present investigation to be correlated with changes in GABA levels as well as in volumetric analysis. As an example, left parahippocampal gyrus connectivity with the r-pIns was negatively correlated with r-pIns GABA concentrations, and right hippocampal connectivity to the r-pIns was found to be inversely proportional to r-pIns GABA concentrations. These areas, in the control group, also exhibited increased cortical thickness post-treatment that trended toward significance, suggesting involvement of the left parahippocampal gyrus and right hippocampus in fibromyalgia-related pain experiences and the ability to modulate these experiences using ST. Reduced hippocampal volume has been found in patients experiencing chronic pain and depression, so an increase in hippocampal volume immediately post-treatment may indicate a return towards a healthy state, correlated with decreased pain. (Mokhtari et al., 2019).

We speculate that the biological pathway underlying auricular neuromodulation resulting in cortical thickness changes is likely related to trans-auricular vagal nerve stimulation (taVNS). Auricular stimulation of the vagus nerve produces significant activation of the central vagal projections, which include widespread activity of cortical and subcortical nuclei such as the locus coeruleus, nucleus tractus solitarius, parabrachial nucleus, anterior and posterior cingulate cortex, amygdala, hypothalamus, insula, thalamus, prefrontal cortex, and primary somatosensory cortex. (Frangos et al., 2015, Zhu et al., 2021) Vagal activation of these various brain regions has been shown to be associated with changes in gray matter volume that correlate with treatment response in studies of epilepsy and depression. (Zhu et al., 2021, Grayston et al., 2019, Perini et al., 2017, Lam et al., 2021).

In the present investigation, we found that cortical thickness significantly decreased in the left middle posterior cingulate (p = 0.018) and increased in the left cuneus (p = 0.014) following ST + PENFS treatment. The posterior cingulate cortex has been related to pain catastrophizing in fibromyalgia, (Lee et al., 2018) and a decrease in volume may suggest a decrease in self-referential pain catastrophizing with ST + PENFS treatment. The posterior cingulate is a part of attentional networks and may be related to the degree to which one is focused on the experience of pain. The cuneus has been related to multisensory integration and cognitive processing including attention, learning, and memory. (Price, 2000) While the left cuneus has previously been shown to be reduced in trigeminal neuralgia, (Parise et al., 2014) its increase following ST + PENFS treatment in our current study of veterans with fibromyalgia has unclear implications, though it may correlate to changes in emotional valence and response to pain.

The cuneus is classically associated with visual information processing, but it also seems to be involved with integration of the somatosensory information with other sensory stimuli and cognitive processes such as attention, learning, and memory (Parise et al., 2014). Reduction in cuneus thickness has been previously demonstrated in patients with trigeminal neuralgia and has been postulated to be related to pain in response to pricking sensation generated by a thermal painful stimulus in trigeminal and extra-trigeminal territory and after selective stimulation of Aδ fibers (Parise et al., 2014). Aδ fibers are thin myelinated fibers that carry cold, pressure, and acute pain signals and often times patients with fibromyalgia are found to have structural abnormalities of these small nerve fibers. The increase in volume of the left cuneus following ST + PENFS treatment could be associated with alterations in signaling via the Aδ fibers and may suggest a reduction in small fiber neuropathy in patients with fibromyalgia.

Concentrations of neurometabolites (e.g. GABA) and gray matter cortical thickness may serve as potential predictors of pain and treatment response, as indicated by the differences in these biomarkers between treatment groups and the correlations with pain scores that were observed in this preliminary study. Though further investigations are needed, there is a potential to develop neuroimaging as an advanced biomarker for the diagnosis of chronic pain syndromes and measurement of treatment response.

**Funding Sources**: This research was supported in part by the US Department of Veterans Affairs Rehabilitation Research and Development Service Career Development Awards 1IK1RX002113-01A2, 1IK2RX003227-01 (Anna Woodbury), and by Senior Research Career Scientist Award Grant B6364L (Bruce Crosson) and Center Grant 5I50RX002358.

## Disclaimer

The views expressed in this article are those of the authors and do not necessarily reflect the position or policy of the Department of Veterans Affairs or the US government.

## Trial registration

Trial registration US National Institutes of Health ClinicalTrials.gov Id: NCT03008837.

## Declaration of Competing Interest

The authors declare that they have no known competing financial interests or personal relationships that could have appeared to influence the work reported in this paper.
